# In vitro antioxidant properties of golden grass (*Syngonanthus nitens*) by electron paramagnetic resonance

**DOI:** 10.1002/fsn3.969

**Published:** 2019-02-14

**Authors:** Rafael P. Barroso, Leonardo S. Berlim, Amando S. Ito, Antonio J. Costa‐Filho

**Affiliations:** ^1^ Departamento de Física, Faculdade de Filosofia Ciências e Letras de Ribeirão Preto Ribeirão Preto Brazil

**Keywords:** antioxidant activity, DPPH, electron paramagnetic resonance, isoorientin, luteolin, *Syngonanthus nitens*, vitamin C equivalent antioxidant capacity

## Abstract

The in vitro antioxidant properties of golden grass (GG), a grass‐like herb (*Syngonanthus nitens*), were investigated by electron paramagnetic resonance (EPR) spectroscopy. We measured the antioxidant capacity of methanolic extracts based on their ability to scavenge 1,1‐diphenyl‐2‐picrylhydrazyl (DPPH) radical. The kinetics of reaction between DPPH and GG extract was determined. This kinetics followed a biexponential decay, and this behavior was attributed to different flavonoids acting together as antioxidants. Isoorientin and luteolin, which are two of the eight flavonoids found in GG extract, were used to investigate kinetics of reaction between DPPH and both the flavonoids acting separately and together. The antioxidant activity of GG extract was determined in terms of the vitamin C equivalent antioxidant capacity (VCEAC). Compared to other well‐known plant‐based antioxidants, such as pulp and peels of fruit and vegetables, *S. nitens* presented a high antioxidant capacity (VCEAC = 1,485 ± 198 mg/100 g), indicating that it should be regarded as a valuable source of antioxidants and also that it may bestow health benefits when consumed.

## 1 INTRODUCTION

Free radicals are atoms or molecules with unpaired electrons that are highly unstable and active toward chemical reactions with other molecules. They are constantly created from cell metabolism and can lead to damage to proteins, lipids, RNA, and DNA, among other molecules (Chan, Gan, & Corke, 2016). An excess of free radicals is related to various severe diseases, including cancer, atherosclerosis, stroke, Alzheimer's, and Parkinson's, among others (Aruoma, 1998; Carocho & Ferreira, 2013). Antioxidants are substances able to prevent or inhibit oxidation processes in human body as well as in food product (Duda‐Chodak & Tarko, 2007). Free radicals can be inhibited by antioxidants.

Antioxidant activities of vegetables and fruits have been evaluated by a wide range of methods (Karadag, Ozcelik, & Saner, 2009; Thaipong, Boonprakob, Crosby, Cisneros‐Zevallos, & Byrne, 2006), such as ferric reducing antioxidant power (FRAP) (Ou, Huang, Hampsch‐Woodill, Flanagan, & Deemer, 2002; Tan et al., 2014), oxygen radical absorption capacity (ORAC) (García‐Ruiz et al., 2017; Sueishi et al., 2012), cupric ion reducing antioxidant capacity (CUPRAC) (Apak, Güçlü, Özyürek, & Karademir, 2004; Tufan, Çelik, Özyürek, Güçlü, & Apak, 2013), total radical‐trapping antioxidant parameter (TRAP) (Pellegrini et al., 2003), 2,2‐azino‐bis(3‐ethylbenzothiazoline‐6‐sulfonic acid) (ABTS) (Di Mattia, Sacchetti, Mastrocola, & Serafini, 2017; Re et al., 1999), and 2,2‐diphenyl‐1‐picrylhydrazyl (DPPH) (Bartoszek, Polak, & Chorążewski, 2017; Thaipong et al., 2006). However, the antioxidant activity cannot be accurately and quantitatively measured by a simple universal method. To directly detect free radicals, EPR spectroscopy is the only analytical technique available. EPR methods have been successfully applied to the determination of antioxidant activity of fruits, herbs (Santos et al., 2009), propolis (Pazin et al., 2017), teas (Popa, Raita, & Toloman, 2015), and spirits (Bartoszek & Polak, 2012; Polak, Bartoszek, & Stanimirova, 2013).

The antioxidant capacity is determined by measuring changes in the EPR spectrum of stable radicals, such as DPPH, as a consequence of their interaction with antioxidants (Zang et al., 2017). DPPH has a maximum UV‐vis absorption within the range of 515–520 nm, presenting a purple color in solution. The radical solution becomes discolored upon reduction, and the reaction progress can be monitored by a UV‐vis spectrophotometer (Brand‐Williams, Cuvelier, & Berset, 1995). However, in the case of antioxidant constituents whose spectra overlap DPPH at its maximum absorbance, the use of EPR spectroscopy is preferred, since it measures the free radical concentration directly (Locatelli et al., 2009). In addition, the decrease in the maximum UV/vis spectrum does not always result in the decrease of the free radical concentration (Yordanov, 1996). Thus, the EPR methodology seems to be more effective to determine the antioxidant activity.

Natural antioxidants occur in all higher plants and in all parts of the plant and are typically phenolic or polyphenolic compounds, including phenols, phenolic acids, flavonoids, tannins, and lignins (Pietta, 2000; Pratt, 1992). In particular, due to their strong capacity to donate electrons or hydrogen atoms, most flavonoids outperform well‐known antioxidants, such as vitamins C and E, in in vitro assays (Hernández, Alegre, Van Breusegem, & Munné‐Bosch, 2009).

Many beneficial activities of flavonoids have been described, for example the use as powerful antioxidants against many diseases including cancers, tumors, allergies, and different free radical‐mediated disorders (Sengupta, Banerjee, & Sengupta, 2005). There is significant increase in using of flavonoids as food additives for health purposes (Tsuchiya, 2010), and other studies indicate that the consumption of foods and beverages rich in flavonoids is correlated with the lower risk of certain cancers, cardiovascular diseases, and oxidative stress‐related diseases (Sisa, Bonnet, Ferreira, & Van der Westhuizen, 2010).

Substances rich in flavonoids are good antioxidant candidates. This is the case of the Brazilian native herb‐like *Syngonanthus nitens* (Bong.) Ruhland, called the golden grass (GG) due to its flower stem or scape that shine like spun gold (Schmidt, Figueiredo, & Scariot, 2007). Their peculiar optical properties are related to the presence of several flavonoids at the surface epidermis of the dry scapes (Berlim et al., 2014). GG stem flavonoids were described as being composed by three flavanones and five flavones (Pacifico et al., 2011). Nowadays, the extract of *S. nitens* stems has been studied as a natural product against different Candida species and clinical isolates from patients with vulvovaginal candidiasis (VVC), which results demonstrated that the antifungal properties are effective and it has the ability to inhibit the fungal‐host invasion on human cells (de Freitas Araújo et al., 2013; dos Santos Ramos et al., 2016). Recently, a work suggested that the study of crude extracts can provide explanations about the compounds behaviors and it shows the importance of possible bioinspired applications of a complete scientific understanding of *S. nitens* (Berlim et al., 2018). Despite the considerable amount of flavonoids in the composition of GG, low attention has been paid to their possible antioxidant properties. In this context, this study aims to investigate the antioxidant properties of the GG.

## 2 MATERIAL AND METHODS

### 2.1 Material

DPPH was purchased from Sigma. Ascorbic acid (AA) was purchased from Panreac (Barcelona, Spain) and used as an antioxidant standard to quantify the antioxidant capacity of GG. Methanol, isoorientin, and luteolin were purchased from Sigma. All reagents were used without further purification. GG was obtained from commercial decorative items available on the market. Fruits used in this study were bought from local market.

### 2.2 Sample preparation

In a plastic tube, an aliquot of 20 µl of methanol (control), AA, isoorientin, luteolin, tangerine extract, or GG extract (samples) was mixed with 20 µl of 1 mM DPPH solution. Immediately after mixing, 25 µl of the homogenate was transferred to a glass capillary tube which was sealed with wax and accommodated within a standard EPR quartz tube. This was in turn placed in the resonance cavity of the EPR spectrometer.

### 2.3 EPR measurement and data analyses

Electron paramagnetic resonance measurements were performed with a JEOL FA‐200 X‐band spectrometer (JEOL Ltd., Tokyo, Japan) at room temperature (21°C). The acquisition conditions were as follows: field modulation frequency, 100 kHz; field modulation amplitude, 1.2 G; sweep width, 80 G; and microwave power, 2.3 mW. Data presented here are an average of six experiments and the uncertainties are the respective standard deviations. The analyses were carried out using the Microcal Origin 7 software.

### 2.4 GG extraction

The extracts of GG were obtained suspending the scapes in methanol (1 g of dried GG per 150 ml) for 24 hr at 25°C and low pressure. After this procedure, the extract passed by a Qualy filter paper, with 80 g/m^2^ of paper weight, 205 µm of thickness, pores with 14 µm, and air permeability at 20 mmCa of 14 L/m^2^s.

### 2.5 Isoorientin and luteolin solutions

Both flavonoids were dissolved in methanol at the desired concentration.

### 2.6 Fruits extraction

Tangerine (*Citrus reticulata* Blanco) was peeled and the peels were blended with water in a hand mixer. The mixture was dried by transferring it to a plastic tube and centrifuged for 7 hr at low pressure (Savant Speedvac plus‐Thermo Quest). Part of the powder was weighted and transferred to another tube to which was added methanol in a proportion of 3.61 mg of powder to 1 ml of methanol. The tube was incubated at room temperature, under slow mixing, for 2 hr. After that, it was centrifuged for 20 min at 16,100 *g* and the supernatant was used as the fruit extract. The same procedure was used to make methanolic extracts of pulp and peels of banana (*Musa acuminata*).

### 2.7 Antioxidant capacity

The area of the integrated EPR spectrum was referred to as EPR signal. The reduction of DPPH EPR signal was calculated using the following equation:

(1) $$I\left(\%\right)=100\times \left(S_{\text{DPPH}}-S_{\text{sample}}\right)/S_{\text{DPPH}}$$

where *I* correspond to EPR signal reduction, and *S*_DPPH_ and *S*_sample_ correspond, respectively, to the EPR signals for solution containing 0.5 mM DPPH in the absence (control) and presence of the methanolic extract (sample).

**Figure 1:**
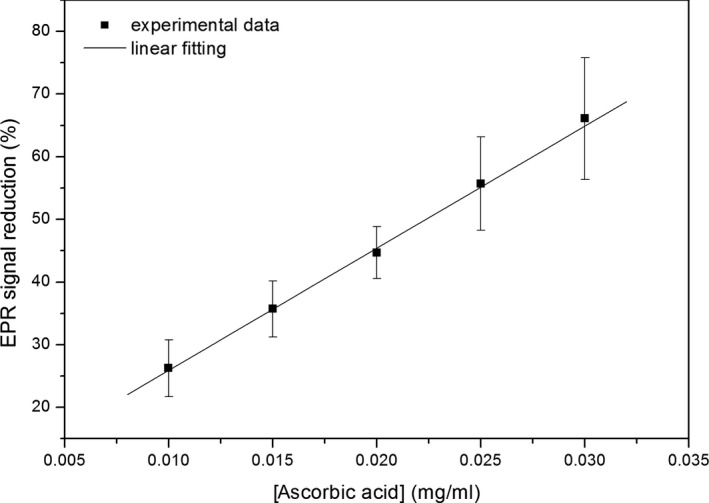
Standard curve: reduction of the electron paramagnetic resonance (EPR) spectrum of 2,2‐diphenyl‐1‐picrylhydrazyl (DPPH) as a function of the ascorbic acid concentration. Fitting parameters: *y* = 6.40 + 1,949.88*x*;* R* ^2^ = 0.998

The antioxidant capacity was expressed in terms of the vitamin C equivalent antioxidant capacity (VCEAC), defined as the amount in mg of ascorbic acid (vitamin C), which causes the same reduction as 100 g of the dry extract (Kim, Lee, Lee, & Lee, 2002). In order to calculate the VCEAC, we first obtained a standard curve by measuring reduction of 0.5 mM DPPH by ascorbic acid with concentrations in a range from 0.01 to 0.03 mg/ml (Figure 1). Using Equation (1), we obtained the reduction caused by a volume of 12.5 µl of GG extract at 0.22 mg/ml concentration and then calculated the equivalent reduction of 100 g of extract. This equivalent reduction was used in the linear equation to then get the ascorbic acid concentration, in mg/ml, needed to produce the same reduction as 100 g of the extract. Finally, we obtained the VCEAC multiplying this concentration by the volume used of ascorbic acid (12.5 µl).

## 3 RESULTS AND DISCUSSION

**Figure 2:**
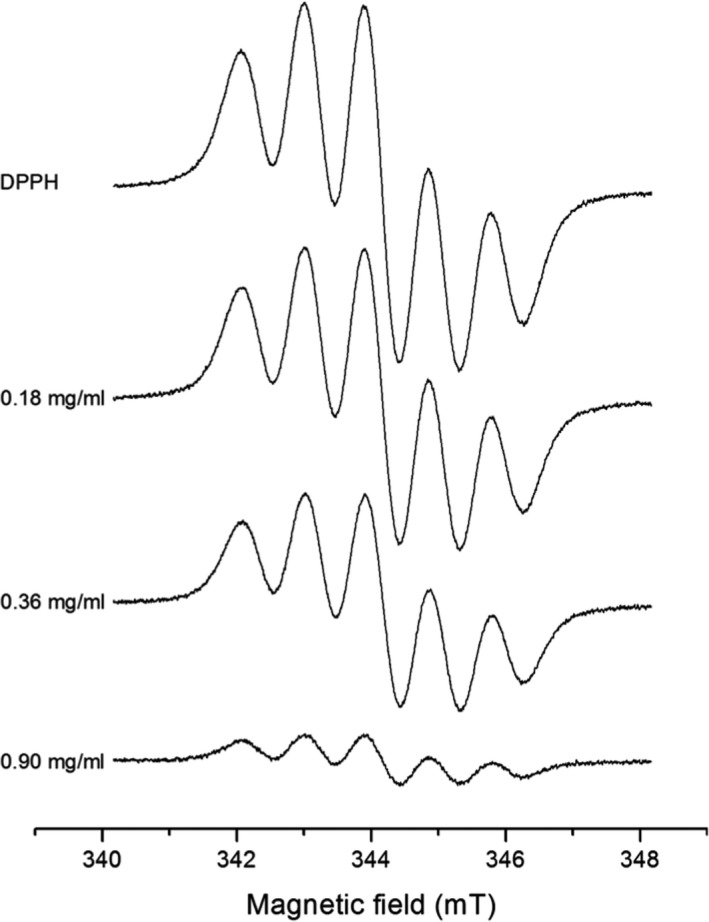
The effect of different concentrations of golden grass on the EPR spectra of DPPH

Figure 2 shows the EPR spectra of DPPH dissolved in methanol in the presence and in the absence of GG extract. The spectra are characterized by five lines due to the interaction of the two equivalent nitrogen nuclei (*g* = 2.0036) with the unpaired electron. The decrease in the line intensity of the EPR spectrum with the increase in GG concentration indicates that there is a chemical reaction occurring between DPPH and the extract. This reaction clearly shows that GG extract presents an antioxidant effect.

**Figure 3:**
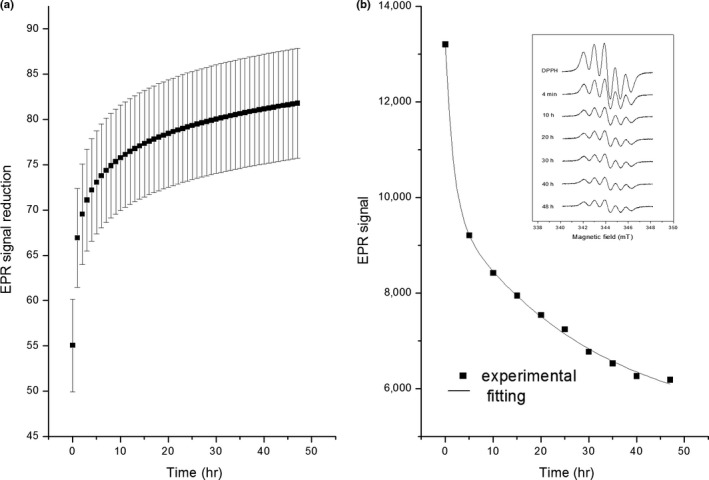
Time effect in the reaction of DPPH with golden grass. (a) Time dependence of the average of EPR signal reduction of five different samples of golden grass. (b) Time decay of EPR signal of DPPH with golden grass. Inset: DPPH spectrum with golden grass along time

The spectra presented in Figure 2 were measured right after mixing the DPPH solution with the GG extract. However, the reaction does not occur instantaneously. To monitor the time evolution of the reaction, the EPR spectrum of DPPH with a GG extract was measured along 48 hr, and the respective EPR signal reduction was calculated along this time. Figure 3a presents the average of six different samples, resulting in error bars around 10% for each time. Considering these error bars, we must wait for at least 3 hr to have a reliable value of the EPR signal reduction and, consequently, to quantify the antioxidant effect properly. It is important to emphasize that in most DPPH assays reported in the literature, the antioxidant activity is measured after an incubation time between 15 min and 2 hr. In a recent work, Fadda et al. stressed this point, drawing attention to the fact that the use of short reaction times may provide underestimated values of the antioxidant activity (Fadda et al., 2014).

The stable free radical DPPH behaves as an electron acceptor from antioxidants (HA). The reaction between DPPH and an antioxidant can be described by the follow equations (Brand‐Williams et al., 1995).

(2) $${}^{{}^{∙}}\text{DPPH}+\text{HA}↔\text{DPPH}-H+{}^{{}^{∙}}A$$

(3) $${}^{{}^{∙}}\text{DPPH}+A^{-}↔{\text{DPPH}}^{-}+{}^{{}^{∙}}A$$

(4) $${}^{{}^{∙}}A+{}^{{}^{∙}}X\to \text{nonradical products}$$

The radicals ^·^A sometimes can be seen by EPR. However, due to the vanishing of these radicals via disproportionation or recombination, usually the concentration of ^·^A is low and therefore they cannot be observed by EPR spectroscopy.

The reduction of DPPH at low concentrations occurs with pseudo‐first‐order kinetics, and this result depends on constituents of the extract. The data presented in Figure 3 follow a first‐order decay until ca 15 hr. However, in order to include all the points in the curve, we had to fit the EPR signal decrease using a biexponential exponential decay described by the equation:

(5) $${\text{EPR}}_{\text{signal}}=S_{0}+\;A\times \text{exp}\left(-t/t_{1}\right)+\;B\times \exp\left(-t/t_{2}\right)$$

**Figure 4:**
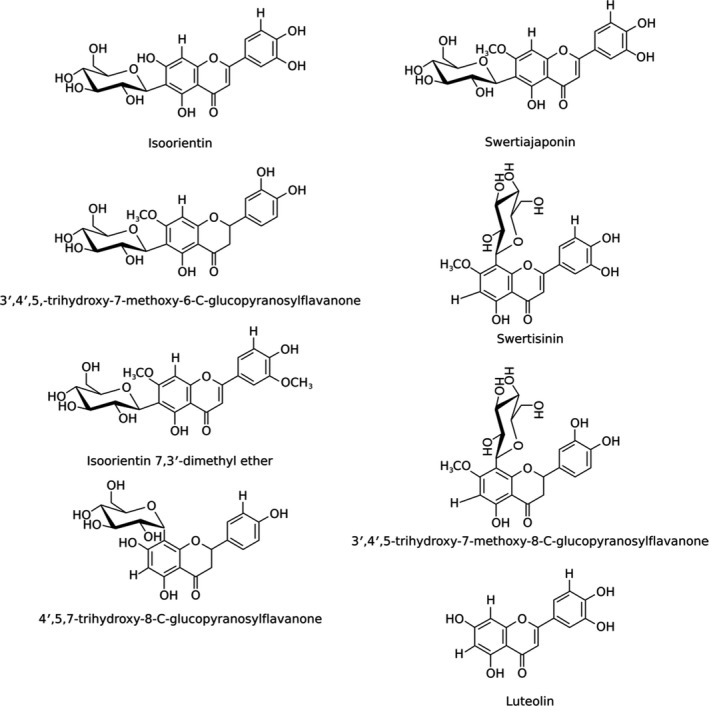
Molecular structure of the flavonoids contained in the golden grass extract

This behavior is compatible with the action of more than one antioxidant substance with different kinetic rates and also is compatible with a nonpurified extract. The GG stem extract is composed by eight flavonoids, five of which are flavones and the other three are flavanones. Their structures and names are presented in Figure 4.

**Figure 5:**
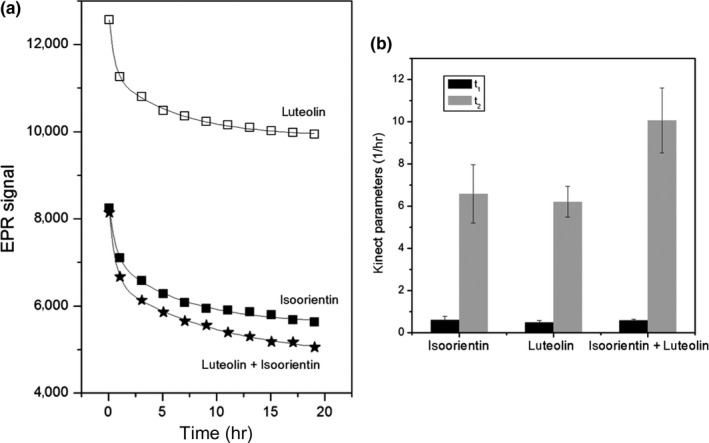
(a) EPR signal kinetic of luteolin and isoorientin acting on DPPH separately and together. Molar ratio DPPH:flavonoid = 10:1 (separately) and DPPH:isoorientin:luteolin = 10:0.5:0.5 (together). (b) Kinetic parameters of biexponential fitting, *S* = *S*_0_ + *A* × exp(−*t*/*t*_1_) + *B* × exp(−*t*/*t*_2_)

Except by luteolin, all the flavonoids have glucose at carbon position 6 or 8. Generally, the antioxidant activity increases with the number of hydroxyl groups and decreases with the number of glycosylation. Phenolic compounds with similar structures exhibit comparable trends in antioxidant activity (Fukumoto & Mazza, 2000). Following this rule, luteolin and swertisin should present the higher and lower antioxidant effect, respectively, and all the other flavonoids should present a similar antioxidant effect, since they have the same number of hydroxyl groups and glycosylation. However, despite the similarity between their structures, Choi et al. showed that the C‐glycosylation position in luteolin affects its antioxidant activity. Indeed, their results showed that isoorientin had four times higher scavenging activity against DPPH than luteolin (Choi et al., 2014). As a very simplified model for a methanolic extract containing more than one antioxidant, we used a solution containing isoorientin and luteolin. We measured the time effect on the DPPH scavenging by both flavonoids acting together and separately (Figure 5a). Isoorientin presented an antioxidant activity 40% higher than luteolin, in qualitative agreement with the results obtained by Choi et al. The EPR signal decrease caused by both isoorientin and luteolin, acting separately and together, also followed a biexponential exponential decay (until ca 3 hr we could fit a first‐order decay for both flavonoids). When acting separately, isoorientin and luteolin have similar values for *t*_1_ and *t*_2_. However, when they acted together, there was an increase in *t*_2_, and the decay became sharper than when the flavonoids were acting on DPPH separately. The concentration of flavonoids acting together was 0.05 mM of luteolin and 0.05 mM of isoorientin, while the flavonoid concentration was 0.1 mM when they were acting independently. This points to a synergic effect of luteolin and isoorientin. In the GG extract, the amount of each of the 8 flavonoids is different, and certainly each flavonoid may contribute differently for the antioxidant effect of GG. We also expect a synergism of all flavonoids leading to an increase in the antioxidant activity of GG. Finally, our simple model can corroborate our hypothesis for the kinetic behavior of the DPPH scavenging by GG extract.

Although the antioxidant activity of chemicals or foods is usually expressed in terms of Trolox (6‐hydroxy‐2,5,7,8‐tetramethylchroman‐2‐carboxylic acid) equivalent antioxidant capacity (TEAC) or IC_50_ values based on molar units, these results are difficult to understand, in particular to nonexperts, leading several authors to suggest VCEAC as a more adequate way to express the antioxidant capacity (Kim & Lee, 2004; Kim et al., 2002). We obtained the VCEAC for extracts of GG, pulp and peels of banana, and tangerine, as shown in Table 1. Flavonoids are one of the most important compounds in the citrus fruits, and several studies have shown that the antioxidant activity of fruits is higher in peel than pulp (Guo et al., 2003; Shin, 2012). In this context, we used extract of tangerine peels as a pattern of high antioxidant activity, as well as a tool to validate the method. In addition, we also measured the VCEAC of banana pulp, which should present a low antioxidant activity.

**Table 1:** Antioxidant capacity of plant‐based antioxidants measured as vitamin C equivalent antioxidant capacity (VCEAC)

Extract	VCEAC (mg/100 g)
Golden grass	1,485 ± 198
Banana pulp	165 ± 111
Banana peel	883 ± 150
Tangerine peel	913 ± 84

We measured the EPR signal decay along time, in the same way showed in Figure 3, and fitted the curves using a biexponential exponential decay (not shown). For each fitting, we obtained the EPR signal, denoted by *S*_sample_ in Equation (1), by extrapolating time to infinity, and calculated VCEAC as described in the section 2.5.

Golden grass extract presented VCEAC higher than tangerine peels, banana pulp and peel, and other plant‐based antioxidants. As expected, the peels of banana present a higher antioxidant activity than the pulp, in agreement with reported by other authors (Guo et al., 2003). In a comparative study of antioxidant activity of 17 common herbs, chamomile presented the highest antioxidant activity with 916 mg VCEAC/100 g (Yoo, Lee, Lee, Moon, & Lee, 2008). In another study, with twenty‐one fruits, Zang et al. obtained a VCEAC range from 11 to 509 mg/100 g (Zang et al., 2017). Thus, GG can be denoted as a good plant‐based antioxidant.

Araújo et al. demonstrated that the methanolic extract of scapes from *S. nitens* is a natural product with antifungal properties against several species of *Candida* (de Freitas Araújo et al., 2013). We speculate that the high antioxidant activity of GG could be related to this antifungal effect and this relation should be investigated.

## 4 CONCLUSIONS

In this study, the antioxidants properties of the GG were investigated for the first time. We showed that methanolic extracts of the stems of GG exhibit a high antioxidant activity, expressed by a VCEAC of 1,485 ± 198 mg/100 g. We attributed this high antioxidant activity to the several flavonoids present in the surface of the stem. GG should be regarded as a valuable source of antioxidants, and it may probably bestow health benefits when consumed. Tea industry could explore the peculiar properties of GG for two reasons. First, its high antioxidant activity contributes to a healthy appeal. Second, the glycoside flavonoids compounds of GG give it a natural sweet flavor. These two reasons point to GG as a good candidate for becoming a popular tea.

## ETHICAL STATEMENT

Human and animal testing is unnecessary in this study.

## CONFLICT OF INTEREST

Authors declare no conflict of interest.
